# Three discipline collaborative radiation therapy special debate: All head and neck cancer patients with intact tumors/nodes should have scheduled adaptive replanning performed at least once during the course of radiotherapy

**DOI:** 10.1002/acm2.12587

**Published:** 2019-04-15

**Authors:** Emilie Soisson, Patrizia Guerrieri, Sundaravadivel Balasubramanian, Anesa Ahamad, Jean M. Moran, Michael C. Joiner, Michael Dominello, Jay Burmeister

**Affiliations:** ^1^ Department of Radiology University of Vermont Burlington VT USA; ^2^ Medical Physics Unit McGill University Montreal QC Canada; ^3^ Department of Radiation Oncology Allegheny Health Network Pittsburgh PA USA; ^4^ Department of Radiation Oncology Medical University of South Carolina Charleston SC USA; ^5^ Department of Radiation Oncology University of Miami Miller School of Medicine Sylvester Comprehensive Cancer Center Miami FL USA; ^6^ Department of Radiation Oncology University of Michigan Ann Arbor MI USA; ^7^ Department of Oncology Wayne State University School of Medicine Detroit MI USA; ^8^ Gershenson Radiation Oncology Center Barbara Ann Karmanos Cancer Institute Detroit MI USA

## 1 INTRODUCTION

Radiation Oncology is a highly multidisciplinary medical specialty, drawing significantly from three scientific disciplines ‐ medicine, physics, and biology. As a result, discussion of or changes in practice within radiation oncology involves input from all three disciplines. For this reason, significant effort has been expended recently to foster collaborative multidisciplinary research in radiation oncology, with substantial demonstrated benefit.^1^, ^2^ In light of these results, we endeavor here to adopt this “team‐science” approach to the traditional debates featured in this journal. This article represents the third in a series of special debates entitled “Three Discipline Collaborative Radiation Therapy (3DCRT)” in which each debate team will include a radiation oncologist, medical physicist, and radiobiologist. We hope that this format will not only be engaging for the readership but will also foster further collaboration in the science and clinical practice of radiation oncology. The advent of Intensity Modulated Radiation Therapy (IMRT) has significantly improved our ability to shape the dose distribution around tumors to spare adjacent normal tissue structures. The treatment of tumors in the head and neck has benefited substantially from this capability since there are typically many adjacent normal tissue structures and both tumors and organs at risk (OARs) tend to be complex in shape. However, creating more conformal treatment plans requires more accurate knowledge of the location and shape of patient anatomy. Anatomical changes throughout the course of treatment can result in significant changes in the delivered dose distribution, thus prompting the creation of new plans during the course of treatment to adapt to these anatomical changes. However, this necessitates a significant increase in workload for radiotherapy staff, increases the cost of care, and provides no guarantee that the anatomy will be the same when the adapted plan is ready to be delivered. Given the potential theoretical improvement in the delivered dose distribution, one may question whether all head and neck cancer patients should receive adaptive replanning. This is the subject of this month's three discipline collaborative radiation therapy debate.

Arguing for the proposition will be Drs. Emilie Soisson, Patrizia Guerrieri, and Sundaravadivel Balasubramanian. Emilie Soisson, PhD, is a medical physicist at the University of Vermont Medical Center. She holds faculty appointments at the University of Vermont and McGill University. She earned a PhD in Medical Physics at the University of Wisconsin where she was heavily involved in the clinical implementation of TomoTherapy, one of the first radiotherapy delivery systems specifically designed for adaptive radiotherapy (ART).

Patrizia Guerrieri, MD, is board certified in Radiation Oncology in Italy and the USA and has an MS in Radiation Sciences. She currently practices at Allegheny Health Network in Pittsburgh and has special expertise in HDR brachytherapy, IMRT, and SBRT, for Head/Neck, Breast, and Gynecological cancers. She has authored publications, abstracts, and book chapters on gynecological brachytherapy, altered fractionation, and brachytherapy in the elderly and was a contributor to the Radiation Oncology Encyclopedia as well as “Principles and Practice of Radiation Oncology” by Perez and Brady.

Sundaravadivel Balasubramanian, PhD, is a cell biology researcher whose primary research focuses on cell signaling pathways altered during radiation treatment in the presence of cigarette smoke and e‐cigarettes, and regulated breathing practices for symptom management in cancer and other conditions. He is also active in teaching radiation biology at the Medical University of South Carolina.

Arguing against the proposition will be Drs. Anesa Ahamad, Jean Moran, and Michael Joiner. Anesa Ahamad, MD, FRCR is an Associate Professor (pending, University of Miami) who trained in Manchester, UK and Houston. She balances patient care with research. With interests in spatial targeting of tumors, combined‐modality tumor‐ablation, and global‐health, she has 138 publications, 27 professional honors and awards, and 115 oral presentations.

Jean M. Moran, PhD, FAAPM is Professor and Co‐Director of the Physics Division in Radiation Oncology at the University of Michigan. Her research areas include patient safety and integration of technology advancements for appropriate patients. She is a Co‐Director of the Michigan Radiation Oncology Quality Consortium, a statewide registry to improve quality. She serves AAPM and ASTRO through committee work.

Michael C. Joiner, MA, PhD, Professor in the Division of Radiation Oncology (Department of Oncology) at Wayne State University, leads WSU's radiobiology research. He focuses on how clinical radiotherapy can be made more effective using manipulations of the radiation delivery schedule. He discovered low‐dose hyper‐radiosensitivity, a major factor influencing the extent and type of signaling and DNA repair following x‐ray exposure and therefore determining overall tumor effect and tissue toxicity.

## 2 OPENING STATEMENTS

### 2.A Emilie Soisson, PhD; Patrizia Guerrieri, MD; Sundaravadivel Balasubramanian, PhD

With the exclusion of early stage true vocal cord cancer, the treatment of head and neck cancers with radiation implies the coverage of many radiation sensitive structures that are either abutting or in close proximity to the treatment volume. Intensity Modulated Radiation Therapy (IMRT, including volumetric modulated arc therapy, and TomoTherapy^®^) has been the standard of care in radiation therapy since the early 2000s and has allowed for the delivery of curative doses of radiation while sparing patients from heavy permanent adverse events at the level of the salivary glands, cervical esophagus, cochlea, spinal cord, glottis pharyngeal muscles, etc. As a result, patients have substantially improved quality of life over the previous 3D conformal era.

A known challenge in head and neck IMRT has been treatment plan robustness over the course of radiation therapy due to anatomical variations over the weeks of treatment, which can lead to increased delivered normal tissue doses over those predicted in the original plan. Radiation therapy impairs swallowing function by causing exudation, inflammation and sloughing of the epithelium which, coupled with the taste impairment typical of the irradiation of salivary glands and oral cavity, is frequently linked to weight loss. Even with IMRT, irradiation of the mucosa cannot be completely avoided due to disease extent, and weight loss must be considered over the course of radiation therapy. In addition, there can be shrinkage of the gross disease or changes of shape or density of the OARs, which also may lead to volume reduction and increased normal tissue doses.

Ultimately, the goal of adaptive replanning is to periodically change the treatment plan so that the delivered dose will more closely resemble the intended planned dose when treatment induced anatomical changes occur. In the case of head and neck cancers, the main advantage is that the normal tissue tolerances can be respected as the tissue volume decreases by accounting for these changes in the treatment plan via incorporation of new imaging data. With the widespread adoption of daily volumetric imaging, these adjustments could also happen as often as daily, making a “plan of the day” possible. In addition, significant technological innovation on the part of the treatment planning system vendors has removed some of the technical hurdles that have made true daily adaptive therapy challenging to implement. Unfortunately, the vendor‐supplied workflows, usually involving automated deformable image registration and dose accumulation, have failed to become the standard of care due to the overhead associated with putting out a new plan, and performing the necessary validation and quality assurance.

While, in theory, delivery accuracy only improves with increased plan frequency, there is limited clinical evidence that daily plan adaptation actually improves dose delivery in a clinically meaningful way for the majority of patients. A prospective multi‐institutional trial by Schwartz et al.^3^ looking at ART for oropharyngeal squamous cell carcinoma showed that the majority of the dosimetric benefits from adaptive planning can actually be achieved with just one or two midtreatment replans.

In fact, the clinical reality at this point is that most centers are using their adaptive planning workflows to perform only one, or a few, adapted plans. Generally, the plan is unanticipated, and a typical workflow follows: A patient is under treatment and at some fraction something or somebody will trigger the need for a new plan. Examples of these triggers vary widely and could be something from a poorly fitting mask, to a physician noticing differences in anatomy upon pretreatment image review, to a more quantitative flag from a more sophisticated “*in vivo*” dosimetry system (i.e., EPID). At this point, in most places, the patient would have to then be scheduled for a repeat simulation with the intent to create a new plan in an accelerated time frame (to avoid treating too many fractions with a potentially invalid plan). In many centers, it means that this plan must take priority over other plans, disrupting the clinical workflow, and altering scheduling or staffing needs. Many steps in the planning and QA may be rushed to allow for the short turnaround time, potentially compromising quality and safety.

The proposition would remove this workflow bottleneck by making an a priori assumption that each head and neck patient will need at least one replan. Creation of an adapted plan at a minimum of one point in a patient's treatment would be easily within the realm of possibility in most places. Automated tools could be used to create the plan, the plan could even be screened using an automated system for dosimetric variation, and then, once flagged, the clinical staff would review this plan for clinical meaningful deviation. After review of this scheduled replan, an informed decision could be made by the clinician to either proceed with the current plan or implement the adapted plan.

Data gathered from this mandated replanning may also make it possible to come up with institution and patient specific predictive factors for anatomical change that will lead to significant dosimetric deviations during treatment. In the future, these adaptive plans could potentially be scheduled at particular time‐points based on the patient's unique risk of requiring one. In addition, scheduled replanning will allow for the incorporation of information regarding treatment response obtained from other image modalities.

It is well known that tumor oxygenation is an important factor in tumor cell kill. The distribution of oxygenated cells within a tumor is nonuniform over time due to changes in tumor vasculature and concomitant hypoxia. 18F‐FDG PET is a widely used tool for mapping tumors based on metabolic activity, hypoxia, and cell proliferation, and has been used for adaptation during radiation therapy.^4^ Recent computer‐based tumor response models suggest that tumor hypoxia based adaptation would be an effective way to reassess treatment dose.^5^ In addition, it has been suggested that a weekly reassessment would be as powerful as daily reassessment where the former could be less cumbersome operationally. While the use of 18F‐FDG PET might be challenging due to the reactivity in areas of radiation‐induced inflammation, this shortcoming could be avoided by using other tracers such as 18F‐FMISO that are hypoxia specific.^6^ Therefore, using 18F‐FDG/18F‐FMISO PET imaging for assessing tumor metabolism, and using that information for IMRT would be an effective way to improve patients’ quality of life as well as reduce normal tissue toxicity. Routine adaptive planning would provide a convenient time point for incorporation of new biological information in the patient's therapy.

In conclusion, we are advocating that all head and neck cancer patients with intact tumors be scheduled for at least one replan over the course of treatment. Scheduled adaptive planning allows for incorporation of anatomical and biological information that can potentially lead to better patient outcomes while allowing for adequate allocation of clinical resources. Timing of replanning events and the necessary plan evaluation criteria could be better informed through new data available as a direct result of implementation.

### 2.B Anesa Ahamad, MD; Jean Moran, PhD; Michael Joiner, PhD

There is a lack of evidence that ART will benefit all head and neck patients and ART could result in protocol deviations. Protocol compliance has been demonstrated to be crucial for improving overall survival.^7^ ART must address the diversity of tumor biology along with anatomical changes and variations in patient setup. For example, patients unlikely to benefit routinely from ART include those with early cancers of the glottis or the skin where only small volumes of tissues are treated. Subjecting all patients to adaptive planning wastes valuable resources. The goal of ART for head and neck cancer is inadequately defined and an emphasis on ART may detract from incorporation of other information such as metabolic pretreatment imaging and treatment considerations.

Routine ART for head and neck cancer patients is just not doable. There is an unmet need for robust and efficient tools for existing equipment which are available and able to be safely used in the broader community for patient care. Investigators have retrospectively demonstrated a correlation of changes on daily cone‐beam CT (CBCT) to xerostomia using a cumbersome process.^8^

Treatment time and the time between fractions are relevant for radiobiology. Thus, minimizing delivery time per fraction can improve effective dose on the tumor by as much as 10%, whilst avoiding more than one fraction per day can minimize toxicity.^9^ While tools are under development to make routine ART feasible, they are not yet perfected. Requirements include fast online imaging, accurate image registration and auto‐segmentation tools, automated quality checks to ensure adequate review of contours, fast dose calculations, and decision support tools, incorporating knowledge of concurrent therapies such as sensitizers, to guide decisions about whether to implement a plan change. The accuracy of image registration must be assessed 10 and this is currently done by humans. Even with validated atlas‐based auto‐segmentation software, review and manual correction of target volumes and normal organs contours is still needed.^9^ The tools for ART are not yet robust, timely, and widely available.

While dose‐reduction to regions where the tumor has regressed in HPV+ oropharyngeal cancers is being considered, its safety, in terms of cancer control or toxicity outcomes, has not yet been validated. Further, the calculated reduction of probability of late effects with decreased dose is quite small.^11^ Dose or volume reduction raises concern about leaving occult disease untreated. It is not clear if tumor resolution on CT, MR or metabolic imaging corresponds to absence of tumor cells. Neither of these three modalities were shown to accurately predict pathologic complete response when studied in patients with breast cancer who were imaged following neoadjuvant chemotherapy and proceeded to surgery (FDG PET/CT shows sensitivity of 38%–89% and specificity of 74%–100%, MRI shows sensitivity of 35%–37% and specificity of 87%–89%).^12^ In order to select patients with breast cancer for de‐escalation of treatment by omitting surgery after neoadjuvant chemotherapy, the only satisfactory technique to determine absence of viable tumor cells was adequate sampling of the tumor bed using stereotactic image guided biopsies.^13^ Therefore, by analogy with breast cancer, it may not be safe to shrink volumes or de‐escalate dose in head and neck cancer without highly accurate verification of absence of tumor cells.

There are far too many unanswered questions to justify routine ART. What is the ideal imaging modality? It is unclear whether adaptation should be based on CBCT, MRI, or metabolic imaging. Which patients would benefit from ART in head and neck cancer?^14^ What is the optimal timing? Should it be performed every five fractions,^15^ within the first 10 fractions?^16^, ^17^ There are no randomized studies that show clinical benefit. How should treatment be altered when changes are detected? Should we escalate dose to persistent tumor or de‐escalate to regions without detectable tumor? Studies of boosting the residual tumor volumes have shown an unacceptably high rate of severe late toxicity with persistent mucosal ulceration.^18^, ^19^ Therefore, boosting residual tumor to higher doses during the adaptive process may not be ready for routine clinical practice.

Finally, some patients may receive a greater benefit from a nonradiation therapy adaptation such as the addition of hypoxic sensitizers if hypoxia is detected and the use of immunotherapy.

## 3 REBUTTAL

### 3.A Emilie Soisson, PhD; Patrizia Guerrieri, MD; Sundaravadivel Balasubramanian, PhD

We agree with our opponents that there are many unanswered questions regarding the ideal clinical use of ART in the treatment of head and neck cancer. It is also true that there is a lack of evidence to show that ART will benefit all patients while there are also significant clinical hurdles to implementation. However, as these technological hurdles lower, ART is only becoming more “doable”. Routine plan adaptation through the smart implementation of new tools and techniques will provide clinical data required to answer some of these questions while allowing clinicians to gain clinical competency and confidence in their adaptive workflows.

Since ideal patient selection for ART in head and neck cancer is not clear, why not give everybody the opportunity for ART through scheduled replanning for all? The statement we are arguing is only that adaptive replanning will be “scheduled” at least once. It would be expected that not all patients will have enough anatomical or biological change to warrant implementation of a new plan. By having a process in place to evaluate anatomical changes and subsequent dosimetric consequences, we will begin to gather clinical data that can later be used to better inform patient selection.

Recent technological innovation in radiation therapy has been focused almost entirely on reducing the volume of tissue irradiated to high doses. How can you fully realize the potential of these technologies without ART? For example, proton therapy distributions are much less robust than photon therapy in the presence of anatomical variation.^20^ The clinical viability of a technology such as the MR‐LINAC is completely dependent on the ability to adapt plans when anatomical changes are seen.^21^ This will also be true as more and more information regarding tumor biology is provided through novel imaging modalities. This scheduled replan is a good point to “check‐in” on the tumor biology as we begin to embrace precision medicine in radiation therapy through well‐designed clinical trials.

As our opponents point out, one of the main hurdles in the implementation of ART has been the availability of the required clinical resources. However, with the increased adoption of automated tools and improved pretreatment image quality, many steps in the planning and plan evaluation process can be automated. Significant improvements have been made in these tools and many investigators have identified streamlined methods to implement them. Validation of automated deformable dose accumulation has always been challenging but in the scenario we propose, patient specific validation would only be required for patients in which significant anatomical change is detected. Currently, ART IS happening in most clinics but all too often in an inefficient and risky way. If all patient plans were adapted, and the adaptive plan was scheduled, workflow bottlenecks that occur as a result of on‐the‐fly replanning could be avoided.

### 3.B Anesa Ahamad, MD; Jean Moran, PhD; Michael Joiner, PhD

The arguments presented by our opposition clearly demonstrate the impracticality of the notion: while scheduling adaptive planning is a great idea, they emphasize the burden of “overhead associated with putting out a new plan and performing the necessary validation and quality assurance”. These activities are cumbersome and wasteful, if an adaptive plan is not used, without a clear benefit to individual patients. Our colleagues cite an important, small pilot study (Schwartz et al.) that showed minor dosimetric benefits for midtreatment replanning but did not show clinical benefit nor was there scientific justification for the timing of replanning. The ideal timing remains under study including new data emerging from daily on‐treatment MRI imaging.

The biology of the target is critical: the uncertainty of accurately locating the volume of residual viable tumor at the site of replanning is still unanswered. The specificity and sensitivity of 18F‐FDG/18F‐FMISO PET remains under study.

Indeed, we agree it would be ideal to avoid the current unexpected decisions to replan, however as a community we lack the needed capabilities in routine practice to automate the process. The radiotherapy plans and online imaging of all head and neck cancer patients with intact tumors and nodes should be routinely analyzed by an intelligent program that can decide whether adaptive replanning is medically necessary. Until this technology is widely available, we are unable to replace the keen clinical observations by physicians, physicists and therapists that triggers replanning and the input from biologists to address fractionation considerations. Therefore, until more robust tools are available, clinical judgment and lessons learned from clinical trials remain best practice.

## CONFLICT OF INTEREST

The authors declare no conflict of interest.
